# Light-controlling, flexible and transparent ethanol gas sensor based on ZnO nanoparticles for wearable devices

**DOI:** 10.1038/srep11070

**Published:** 2015-06-16

**Authors:** Z. Q. Zheng, J. D. Yao, B. Wang, G. W. Yang

**Affiliations:** 1 State Key Laboratory of Optoelectronic Materials and Technologies, Nanotechnology Research Center, School of Physics & Engineering, Sun Yat-sen University, Guangzhou 510275, Guangdong, P. R. China; 2 Shenzhen Key Lab of Micro-nano Photonic Information Technology, Shenzhen Key Laboratory of Sensor Technology, College of Electronic Science and Technology, Shenzhen University, Shenzhen 518060, Guangdong, P. R. China

## Abstract

In recent years, owing to the significant applications of health monitoring, wearable electronic devices such as smart watches, smart glass and wearable cameras have been growing rapidly. Gas sensor is an important part of wearable electronic devices for detecting pollutant, toxic, and combustible gases. However, in order to apply to wearable electronic devices, the gas sensor needs flexible, transparent, and working at room temperature, which are not available for traditional gas sensors. Here, we for the first time fabricate a light-controlling, flexible, transparentand working at room-temperature ethanol gas sensor by using commercial ZnO nanoparticles. The fabricated sensor not only exhibits fast and excellent photoresponse, but also shows high sensing response to ethanol under UV irradiation. Meanwhile, its transmittance exceeds 62% in the visible spectral range, and the sensing performance keeps the same even bent it at a curvature angle of 90^o^. Additionally, using commercial ZnO nanoparticles provides a facile and low-cost route to fabricate wearable electronic devices.

In recent years, due to the significant applications of health monitoring, wearable electronic devices, such as smart watches, smart glass and wearable cameras have been growing rapidly. More studies have focused ondeveloping of new wearable electronic devices^1^,^2^,^3^. Gas sensor is an important part of wearable electronic devices detecting pollutant, toxic, and combustible gases. Semiconducting metal oxide nanostructures, such as ZnO nanorods^4^, SnO_2_ nanowires^5^ and In_2_O_3_ hollow spheres^6^ have been widely reported to be good candidate gas sensors for highly sensitive and stable due to their high surface-to-volume ratio. Among these gas sensor nanomaterials, ZnO nanostructures have been extensively investigated due to their high conductivity, good stability and biological friendliness^7^,^8^,^9^. Upon exposure to reducing gas such as ethanol, ethanol molecules will reach with the adsorbed oxygen ions on the surface of ZnO nanostructures, which can releases free electrons back to the conduction band of and narrow the depletion width, leading to a great increase in the conductivity of the ZnO nanostructures^7^,^8^,^9^.

Although ZnO nanostructures gas sensors have been widely used in practice, the high working temperature^10^,^11^ and complex structures^12^ is undesirable for wearable electronic devices. In order to apply to wearable electronic devices, the gas sensor needs flexible, transparent, and working at room temperature, which are not available for traditional gas sensors. Therefore, developing the new generation of flexible, transparent and room temperature working gas sensor is a quite attractive and challenge task.

For operating at room temperature, some techniques like noble metals such as Au^13^ and Pd^14^ modified nanomaterials has been confirmed to have potential to greatly enhance the sensitivity and decrease working temperatures of traditional gas sensors. But the high cost seriously limits their applications. Among these techniques, light irradiation attracted increasing attention as a promising strategy to improve the sensing performance^15^, and some reports showed that light irradiation enables sensors to operate at room temperature^16^,^17^,^18^. In terms of flexibility and transparency, among the various interesting materials used for substrates, PET coat ITO (PET-ITO) exhibit excellent dielectric properties, outstanding chemical stability, highly flexible and nearly transparent, have been widely used as flexible transparent substrates^19^,^20^,^21^,^22^.

In this contribution, using a simple and cost effective drop-casting method to coat commercial ZnO nanoparticles on the flexible and transparent PET-ITO substrate, we for the first time fabricate a UV-light controlled, flexible, transparentand working at room-temperature ethanolgas sensor ethanol sensor. Under UV irradiation, this sensor is highly sensitive to ethanol at room temperature. Using PET-ITO support instead of conventional substrates, the transmittance of the sensor is more than 62% over the visible range (400–800 nm). Meanwhile, the sensing performance keeps the same even bent it at a curvature angle of 90°. Thus, these results demonstrate that the fabricated ethanol gas sensor could be expected for applicable to wearable device.

## Results

**Figure 1:**
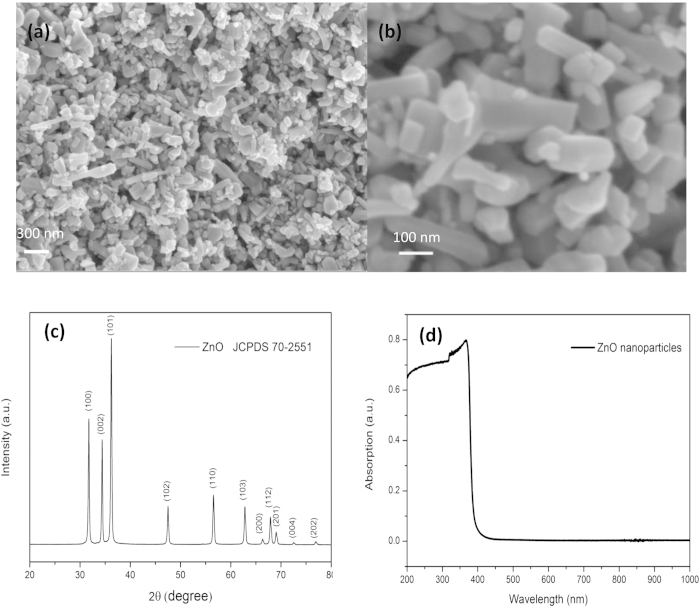
(**a**) The typical FESEM image of ZnO nanoparticles. (**b**) The morphology of ZnO nanoparticles. (**c**) XRD diffraction pattern and the corresponding lattice planes of ZnO nanoparticles. (**d**) UV-vis diffuse reflectance spectra of the sample.

FESEM images of ZnO nanoparticles are shown in Fig. 1a,b. Clearly, the sizes are ranging from tens to one hundred nanometers. The corresponding XRD pattern in Fig. 1c shows that all the diffraction peaks can be indexed as the hexagonal wurtzite ZnO (JCPDS NO. 70-2551), indicating its high purity. UV–vis spectra in Fig. 1d shows the sample only absorbs the light of the wavelength less than 400 nm, and the largest intensity of the absorbance wavelength is distributed around 370 nm. Thus, in order to get the best photoelectric response, monochromatic light with wavelength 370 nm is selected to illuminate the device in our case.

**Figure 2:**
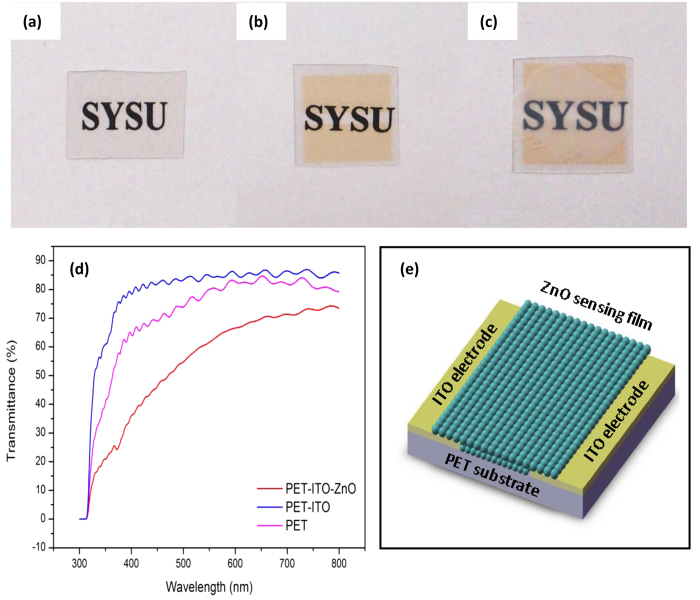
Optical images of (**a**) PET, (**b**) PET-ITO substrate and (**c**) PET-ITO-ZnO device. (**d**) The corresponding optical transmittance spectra. (**e**) The schematic diagram of the sensor.

One interesting aspect of the device is that it exhibits good optical transmittance in the visible spectral range. Fig. 2a–c show the optical images of PET, PET-ITO substrateand the device after ZnO coats on the PET-ITO substrate (PET-ITO-ZnO), respectively. It can be seen that a SYSU logo beneath the transparent device can easily be seen. The corresponding transmittance spectra in Fig. 2d show that the PET substrate exhibits a maximum transmittance of 87% and an average transmittance of 84% over the visible range, and the PET-ITO substrate exhibits a maximum transmittance of 83% and an average transmittance of 78% over the same range. Even after the transfer of ZnO nanoparticles, the average optical transmittance still exceeds 62% in the visible spectral range. Thereby, we can demonstrate it as an invisible gas sensor. Note that that there exists anarea around 370 nm where the optical transmittance decreases, that is because of the largest intensity of absorbance in these wavelengths.

**Figure 3:**
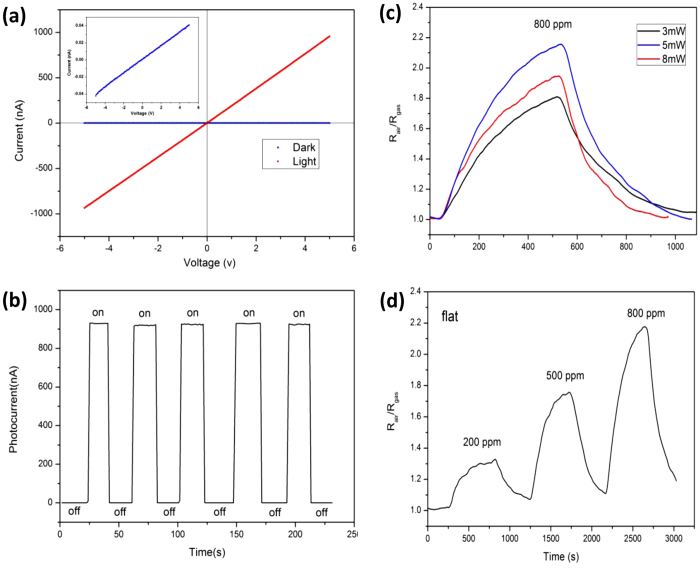
(**a**) I-V characteristics of the sensor in dark and in illuminated with 370 nm light (5 mW), the inset is the higher magnification I-V characteristic under dark condition. (**b**) Time-dependent photoelectric response of the device measured by periodically turning on and off 370 nm light (5 mW) at an applied voltage of 5 V. (**c**) Dynamic response curves of the sensor to 800 ppm ethanol gas at room-temperature under 370 nm light irradiation when the light intensities varies from 3 to 8 mW. (**d**) Dynamic response curve of the device exposure to ethanol concentrations ranging from 200 to 800 ppm under 370 nm light illumination (5 mW) at room-temperature.

Another interesting aspect of the device is that it exhibits highly sensitive to ethanol gas at room temperature under UV irradiation. The I-V characteristics of the sensor, which is measured in dark condition and illuminated with 370** **nm light (5** **mW) at room-temperature is shown in Fig. 3a, and the insert shows the higher magnification I-V characteristic under dark condition. The linear I–V characteristics indicate the good ohmic contacts with no interfacial barrier or traps between the sensing film and ITO electrodes. When the device is illuminated by the 370** **nm UV light at an applied voltage of 5** **V, the current across the device dramatically increases from 0.04 to 930** **nA. The corresponding ratio of photocurrent to dark current of the sensor is as high as 23250, which is significantly better compared with previous reports^23^,^24^. The increase in photocurrent under 370** **nm light illumination can be understood in terms of increased number of excited electron-hole pairs when the photo energy becomes larger than the bandgap of ZnO. Fig. 3b presents the time-dependent photoelectronic response of the device measured by periodically turning on and off 370** **nm light at an applied voltage of 5** **V at room-temperature. Once UV light is on, the photocurrent increase quickly (less than 1** **second) from 0.04** **nA to a stable value of 930** **nA, and then dramatically decreased (also less than 1** **second) to its initial value once the UV light is turned off. The maximum photocurrent of each cycle is nearly the same, and it maintains stable if the UV light is on, showing excellent stability and reproducible characteristics.

As a light-controlling gas sensor, the sensing performance to ethanol gasunder UV light should be investigated. We calculate the response of the sensor using the expression of Response% = R_o_/R_g_^25^, where R_o_ and R_g_ are the resistance of the sensor before and in exposing to ethanol gas, respectively. Fig. 3c shows the dynamic response curves of the ethanol gas sensor to 800 ppm ethanol gas at room-temperature under 370 nm light irradiation at different light intensities varies from 3 to 8 mW at a bias voltage of 8.7 V. We can see that the response obviously increases when the sensor is exposed to ethanol gas, upon purging with clean air, the responses quickly get back to the initial value, and the sensing properties are strong influenced by the light intensity. In order to obtain the optimized sensing performance, the suitable light power (5 mW) is chosen in our experiments. To further confirm the sensing properties of the fabricated gas sensor, the dynamic response curve of the device exposures to different concentrations of ethanol gas ranging from 200 to 800 ppm under 370 nm light illumination (5 mW) at room-temperature are determined. As shown in Fig. 3d, while the concentration of ethanol gas increased from 200 to 800 ppm, the sensor demonstrated responses increases with the higher ethanol concentration level from 1.3 to 2.2. The performance of the sensor is better compared with many reported sensors^16^,^26^,^27^,^28^. It is worth noticing that there is no obvious gas sensing response without light, by contrast, we can demonstrate it as a light-controlling gas sensor.

**Figure 4:**
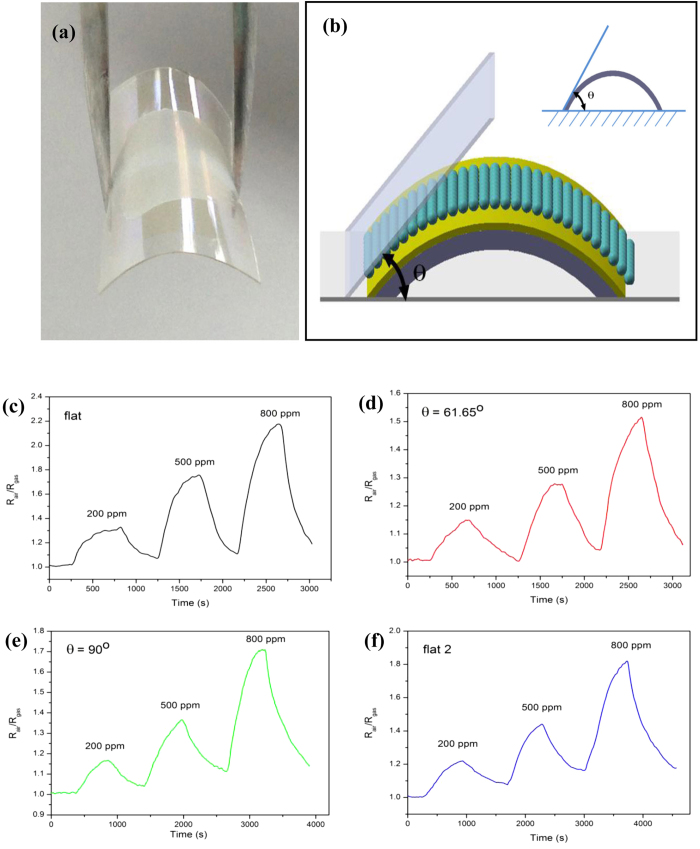
(**a**) Optical image of the fabricated transparent and flexible gas sensor. (**b**) The schematic image of the direction of the bending of the sensor, and the corresponding curvature angle, and the inset shows the cross-section of the sensor. Performance of the sensor under 370 nm light illuminations (5 mW) at room-temperature for various bending angles: (**c**) 0°, (**d**) 61.65°, (**e**) 90°, and (**f**) return to 0°.

As mentioned previously, mechanical flexibility is essential to wearable electronic devices. What is more, flexible sensors own many advantages over rigid sensors with their ability to be conformal over surfaces leading to less occupation of space^29^,^30^. Bending experiment is performed by directly bending the sensor from 0° (flat) to various curvature angles of 61.65° and 90°, the corresponding radius of curvature in the bending state are 5.12** **mm and 3.5** **mm, respectively. Then return the sensor to 0° (flat-2). The optical image of the fabricated transparent and flexible gas sensor is shown in Fig. 4a. And the schematic image of the direction of the bending-induced strain in the sensor is shown in Fig. 4b, and the inset shows the cross-section of the sensor. Fig. 4c–f show the real-time response of the sensor at various concentrations of ethanol gas under 370** **nm light illumination (5** **mW) at room-temperature for relative difference bending angles. As evident, there is basically not change in the response even bending the curvature angle to 90°. Thus, these results clearly indicate that bending of the sensor did not affect its sensing performance.

## Discussion

The light-controlling sensing mechanism is described as follows. When ZnO nanoparticles are exposed to air in the dark, the adsorbed oxygen molecules trapping electrons from the conduction band of ZnO and transferring as O_2_^−^(O_2_** + **e^−** **^**→ **O_2_^−)^^31^ at room-temperature, resulting in the presenceof a low-conductivity depletion region in the surface layer and narrow the conductionchannels in ZnO. As the large adsorption energy, the oxygen ion (O_2_^−^) is thermally stable and difficult to be removed from the ZnO surface at room temperature^32^, and cannot reacted with ethanol molecules. So there is no obvious gas sensing responsein the dark.

When the device is illuminated with 370 nm light, the photo-induced electron-hole pairs will be generated in ZnO due to the larger photon energy than the band gap of ZnO (3.2 eV)^33^. Some of the photo-generated holes will desorb the adsorbed oxygen ions on the surface according to the following reactions: h^+^ + O_2_^−^ → O_2_, resulting in a reduction in the depletion layer width and an increase in the free carrier concentration, which results in dramatically increases photocurrent upon the UV light^24^. On the other hand, with the raised free carrier density, the ambient oxygen molecule reacting with the photo-generated electrons, creating a new photo-induced chemisorption oxygen molecule^16^ as the following scheme: O_2_ + e^−^ (hν) → O_2_^−^ (hν). Unlike the chemisorbed oxygen ions which arestrongly attached to the ZnO surface, these photo-generated oxygen ions [O_2_^−^ (hν)] are weakly bound to ZnO and can be easily removed^32^, which make it hasobvious gas sensing response just at room temperature. When the sensor is exposed to ethanol gas, these additional adsorbed oxygen molecules on the surface of ZnO will reacted with ethanol molecules as the following reactions: C_2_H_5_OH + 3O_2_^−^ (hν) → 2CO_2_ + 3H_2_O + 3e^−^, which release electrons back to the conduction band of ZnO, decreases the surface depletion layer width and then increases electrical conductivity of the device. In other words, the response obviously increases when the sensor is exposed to ethanol gas. Upon purging with clean air, O_2_ molecules gradually readsorbed on the surface and capture the electrons conduction band of ZnO again, which results in a gradual response decay to the initial value. Then the light-controlling adsorption-desorption cycle of oxygen is established. As the carrier density under UV light is much larger than that in the dark, it would the adsorption-desorption more O_2_, and photo-induced oxygen ions [O_2_^−^ (hν) ]are highly reactive and responsible for the room-temperature gas sensitivity^32^, the gas sensing response exhibits much more obvious under light compare with in the dark. In this way, we demonstrate it as a light-controlling gas sensor.

In summary, we have fabricated and UV-light controlled, flexible, transparent and working at room-temperature ethanol gas sensor by simply coating ZnO nanoparticles on flexible and transparent PET-ITO substrates. As the high density of free carrier and highly reactive photo-induced oxygen ions are generated under UV irradiation, this sensor not only shows excellent photoelectric response, but also exhibits remarkable sensitive to ethanol gas at room-temperature with 370 nm light illumination, while the sensor has no response to ethanol gas in the dark. In addition, its sensing performance keeps no change even bent it at a curvature angle of 90^o^. These present results suggested that the fabricated sensor is a good candidate for wearable electronic devices.

## Methods

### Materials and characterization

Commercial ZnO nanoparticles (the size is 40–100** **nm and the surface area ratio is 10–25** **m^2^/g) was purchased from Alfa Aesar, and was directly used as the sensing materials without further purification. Field-emission scanning electron microscopy (FESEM) was used to characterize the morphology of the samples, phase purity and structure of ZnO nanoparticles were characterized by X-ray diffraction (XRD) with Cu Kα radiation scanning from 20–80° at room temperature. The UV-vis diffuse reflectance spectrum and the optical transmittance of were employed by using an ultraviolet–visible–near infrared (UV-Vis-NIR) spectrophotometer, while BaSO_4_ and air were used as a reference, respectively.

### Device fabrication and sensing measurements

The substrate was made of PET coated with ITO (35 Ω/square) on one side surface. Then the ITO had been etched by laser ablation to remove many strips of the conducting coating to form parallel electrodes. The channel width between the two parallel electrodes is 1** **mm, each ITO electrode area was 4** × **9** **mm^2^, and the thickness of ITO was 180** **nm. This sensor was fabricated by a simple drop-costing method. At first, ZnO nanoparticles was uniformly dispersed in deionized (DI) water to produce thin slurry. Secondly, two drops (8** **μl) of the slurry was dropped on the channel between parallel electrodes, and then kept at 70** **°C for 2** **hours to vaporize DI water, then a ethanol sensor base on ZnO NPs was obtained.

The photoelectric and current versus voltage (I-V) curves was studied using a Keithley 4200-SCS semiconductor characterization system. The measurements were conducted in sampling mode at room-temperature and monochromatic light with wavelength 370 nm was used to illuminate the device. Gas sensing properties were measured by the gas sensing characterization system, and 370 nm monochromatic light irradiated on the sensor through the quartz window of the test chamber. Certain concentration of ethanol or clean air was periodical passed into the test chamber based on a flow-through technique. The total flow rate was kept at 500 sccm, and all the measurements above were carried out at room temperature.

## Additional Information

**How to cite this article**: Zheng, Z. Q. *et al.* Light-controlling, flexible and transparent ethanol gas sensor based on ZnO nanoparticles for wearable devices. *Sci. Rep.* **5**, 11070; doi: 10.1038/srep11070 (2015).
